# *Hymyc1* Downregulation Promotes Stem Cell Proliferation in *Hydra vulgaris*

**DOI:** 10.1371/journal.pone.0030660

**Published:** 2012-01-23

**Authors:** Alfredo Ambrosone, Valentina Marchesano, Angela Tino, Bert Hobmayer, Claudia Tortiglione

**Affiliations:** 1 Istituto di Cibernetica “E Caianiello,” Consiglio Nazionale delle Ricerche, Pozzuoli, Italy; 2 Zoological Institute and Center for Molecular Biosciences, University of Innsbruck, Innsbruck, Austria; Institute of Genetics and Molecular and Cellular Biology, France

## Abstract

*Hydra* is a unique model for studying the mechanisms underlying stem cell biology. The activity of the three stem cell lineages structuring its body constantly replenishes mature cells lost due to normal tissue turnover. By a poorly understood mechanism, stem cells are maintained through self-renewal while concomitantly producing differentiated progeny. In vertebrates, one of many genes that participate in regulating stem cell homeostasis is the protooncogene *c-myc*, which has been recently identified also in *Hydra*, and found expressed in the interstitial stem cell lineage. In the present paper, by developing a novel strategy of RNA interference-mediated gene silencing (RNAi) based on an enhanced uptake of small interfering RNAi (siRNA), we provide molecular and biological evidence for an unexpected function of the *Hydra myc* gene *(Hymyc1)* in the homeostasis of the interstitial stem cell lineage. We found that *Hymyc1* inhibition impairs the balance between stem cell self renewal/differentiation, as shown by the accumulation of stem cell intermediate and terminal differentiation products in genetically interfered animals. The identical phenotype induced by the 10058-F4 inhibitor, a disruptor of c-Myc/Max dimerization, demonstrates the specificity of the RNAi approach. We show the kinetic and the reversible feature of *Hymyc1* RNAi, together with the effects displayed on regenerating animals. Our results show the involvement of *Hymyc1* in the control of interstitial stem cell dynamics, provide new clues to decipher the molecular control of the cell and tissue plasticity in *Hydra*, and also provide further insights into the complex *myc* network in higher organisms. The ability of *Hydra* cells to uptake double stranded RNA and to trigger a RNAi response lays the foundations of a comprehensive analysis of the RNAi response in *Hydra* allowing us to track back in the evolution and the origin of this process.

## Introduction

Despite its simple body plan and structural anatomy, the Cnidaria *Hydra*, a dipoblastic animal at the base of metazoan evolution, is an excellent model system to investigate the mechanisms controlling stem cell proliferation and differentiation and the balance between the two phenomena. While in most of animals the self-renewing property is confined to one or more tissues, in *Hydra* most of the cells continuously divide and are displaced towards the animal extremities where terminal differentiation occurs before cell loss. The constant growth process requires a homeostatic regulation within and between different cell lineages and a steady state of production and loss of cells [1]–[4]. The polyp is composed of two epithelial layers, an outer ectoderm and an inner endoderm, shaping a tube-like body, with a single opening (mouth) at one end and a foot to anchor to a substrate at the opposite end. Each layer, which is a single cell deep, comprises a cell lineage. All other cell types are lodged in the interstices among the epithelial cells of both layers, and are part of the interstitial cell lineage (see Figure S1). Interstitial cells occur singly (1 s) or in clusters of 2, 4, 8 and 16 cells (2 s, 4 s, 8 s, 16 s). All these classes are actively proliferating, with a cell cycle length of about 1 day [5]. Single interstitial cells or clusters of 2 form neurons (sensory and ganglion cells) and secretory cells (zymogen and mucous cells), while clusters of 4, 8 and 16 interstitial cells (nematoblasts) differentiate different types of nematocytes. Multipotent interstitial stem cells, which do not differentiate but simply proliferate, must exist to provide for growth of the interstitial cell population and to provide a continuing supply of differentiating nematocytes, nerve cells, gland cells and gametes [6]. Clearly, a variety of control mechanisms are needed to maintain steady state levels of mature cells, as well as to stimulate the rapid production of specific cell types as needed. This might requires the participation of many factors, including positive and negative regulators of growth and differentiation, which determine survival, growth stimulation, growth arrest, differentiation.

In vertebrates, one of many genes that participate in regulating cell homeostasis is the protooncogene *c-myc*. The *MYC* family of transcription factors controls disparate aspects of cell physiology including cell growth, cell cycle progression, biosynthetic metabolism, and apoptosis [7]–[9] and, as expected, its deregulated expression occurs in the majority of human cancers. Recently, in *Hydra magnipapillata*, a *c-myc* homologue (*Hymyc1*) has been isolated and fully characterized [10], and by *in silico* genomic analysis three additional *myc*-like or -related genes have been predicted. Among them, HyMYC1 and HyMYC2 deduced protein sequences display the principal topography of MYC proteins, i.e. bHLH- Zip domains and MYC boxes I to III, and are clearly orthologues of vertebrate *myc*, while the predicted proteins of the two others (HyMYC3 and HyMYC4) present bHLH-Zip domains but are highly divergent in the N-terminal regions, suggesting myc-related roles. *Hymyc1* is expressed in proliferating fractions of the interstitial stem cell system, namely single and pairs of interstitial stem cells, proliferating nematoblasts, gland cells. Recombinant hybrid proteins between *Hymyc1* and viral *myc* genes displayed, in assays of cell transformation, oncogenic potential, suggesting structural and functional conservation of *HyMYC1* protein domains. By contrast, the functional role played in the interstitial stem cell lineage in *Hydra* is unknown. To this aim, we have developed a new RNA interference (RNAi) approach to downregulate *Hymyc1* expression. By using small interfering RNAs (siRNA) [11] specifically designed to target *Hymyc1*, we overcome the main issue of their delivery to target tissues by modifying their highly negative charge towards positive values. We have previously shown the capability of *Hydra* to actively uptake positively charged nanoparticles suspended in the culture medium [12]. Here we show that acidic condition enhances the entry of siRNA duplexes into the polyps triggering the *Hydra* RNAi response and leading to specific post transcriptional *Hymyc1* inhibition. Under normal feeding regime and physiological culturing condition, a large scale screening of RNAi phenotype was possible, while avoiding previously used invasive delivering methods to alter gene expression, such as electroporation [13]–[15]. We provide molecular evidence of *Hymyc1* reduced expression level, while analysis at cellular level led to decipher an unexpected function in the homeostasis of interstitial stem cells. *Hymyc1* inhibition impairs the balance between stem cells self-renewal/differentiation, as shown by the accumulation of intermediate and terminal differentiation products (nematoblasts, nematocytes and secretory cells). The biochemical repression of *Hymyc1* activity achieved by using 10058-F4 inhibitor, a disruptor of c-MYC/MAX dimerization, produced similar effects on *Hydra* polyps, confirming the specificity and the validity of our RNAi approach.

The kinetic of *Hymyc1* RNAi was also evaluated, together with the effect displayed on the regenerative capability of the polyp. Together with the establishment of a reliable loss of function assay to analyse gene function in *Hydra*, the specific *myc* silencing opens new avenues to decipher the molecular control of the cellular plasticity underlying growth and proliferation in *Hydra*, providing further insights into the complex *myc* network also in higher organisms.

## Results

### *Hymyc1* downregulation through siRNA

**Figure 1:**
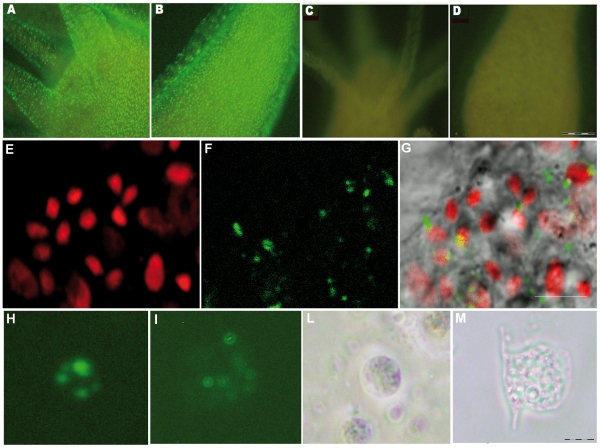
*In vivo* RNAi mediated by siRNA. Fluorescence imaging of *Hydra vulgaris* exposed to *myc*-siRNA at different pHs. Living polyps were challenged with 70 nM Alexa488-end labelled *myc*-siRNA, in *Hydra* medium either at pH 4 (A, B) or at pH 7 (C, D). After 24 hr of continuous incubation with siRNA a strong punctuated fluorescence labels uniformly the whole animal, from the head (A) along the body (B) of animals treated at acidic pH, indicating the uptake of siRNA. In (C) and (D) are shown the body regions corresponding to A and B, respectively, of animals treated with the same siRNA at neutral pH. The absence of fluorescence signals indicates that the acidic pH enhances siRNA delivery in *Hydra*. Scale bar 200 µm. Confocal laser scanning imaging (E–G) of living *Hydra* treated 24 hr with Alexa488-labelled *myc*-siRNA revealed localization of oligonucleotides into interstitial cells. In (E), nuclear staining of interstitial cell nest with TOPO3; in (F) siRNA green fluorescence appears as faint staining or more evident granules; in (G), the overlay of bright field and fluorescence images revealed that siRNAs localize prevalently into the cytoplasm of interstitial cells. Scale bars: 10 µm. Fluorescence microscopy analysis of single cells suspensions prepared from treated dissociated animals shows intracellular localization of siRNA into interstitial (H) and epitheliomuscular (I) cells, as a punctuated pattern. The same cells were observed by phase contrast microscopy, respectively in (L) and (M). Scale bar 10 µm.

**Figure 2:**
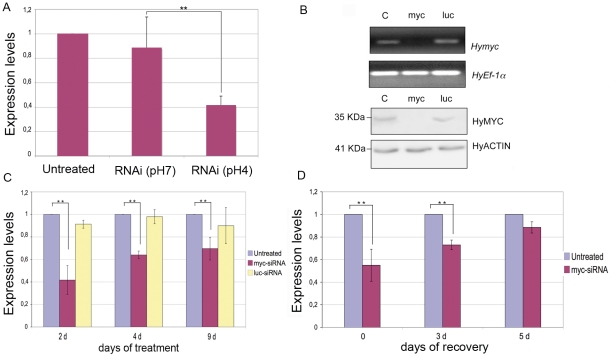
Molecular characterization of *Hymyc1* RNAi. (A) quantitative RT-PCR showing *Hymyc1* downregulation enhanced by acidic condition. Animals treated by *myc*-siRNA at pH 4 showed 60% decrease in *Hymyc1* transcription levels compared to *Hydra* Elongation factor *HyEF1*α mRNA, used as internal control (two asterisks, p<0.01 according to t-Student test). (B) Upper panel: semi-quantitative RT-PCR showing *Hymyc1* downregulation induced specifically by *myc*-siRNA (lane myc) and not by *luc*-siRNA (lane luc), or in untreated animals (lane C) used as controls. Lower panel: *Hymyc1* RNAi affects also MYC protein levels. Lane labels are as in the upper panel. MYC protein levels were detected using anti-HyMYC1 antibody (1∶500, kindly provided by K.Bister, University of Innsbruck) and compared to actin proteins, using an anti-actin primary antibody (1∶100, Sigma) to probe an identical blotted gel. HyMYC1 shows an apparent mol. weight of 35 kDa, as elsewhere reported [10]. (C) Kinetics of *Hymyc1* downregulation. qRT-PCR was performed on total RNA extracted from 25 animals either untreated (namely incubated at pH 4, in absence of siRNA) or incubated with the indicated siRNA for different periods. The most effective downregulation is detected at the beginning of the treatment, and it is specifically induced by *myc*- and not *luc*-siRNA duplexes (two asterisks, p<0.01 according to t-Student test). (D) Reversible effect of RNAi. Animals were treated with *myc*-siRNA for 4 d (time t = 0) and then cultured in physiological condition for the indicated periods of time (3 days, 5 days), when total RNAs were extracted for qRT-PCR analysis. Suspension of RNAi treatment restored in five days *myc* mRNA transcript levels up to physiological values. Error bars in A, C and D indicate standard deviations calculated from three independent experiments, each performed in triplicate.

In a previous study [12] we showed that at acidic pH fluorescent semiconductor nanoparticles, showing positive surface charge, are actively internalized by *Hydra* ectodermal cells, while negatively charged nanoparticles are not uptaken. With the aim to downregulate *Hydra myc1* gene we tested the possibility to perform RNA interference mediated by small interfering RNAs. 21 bp long RNA oligonucleotides with symmetric 2 nt 3′ overhangs targeting the coding region of the *Hymyc1* gene (*myc*-siRNA) were designed according to specific rules found to enhance the silencing effect of siRNA [16]–[18] and chemically synthesized. An estimation of their surface net charge was achieved by zeta potential measurements, a method widely used to quantify the electrokinetic potential in colloidal systems [19]. Zeta potential measurement of siRNA duplexes as function of pH showed that they present positive net charge at pH 4 in *Hydra* culture medium (see Figure S2 of Supporting Information), while at physiological pH, as expected, they are negatively charged. The silencing properties of *myc*-siRNA were thus tested at pH 4, by soaking the animals in their culture medium in presence of 70 nM *myc*-siRNA, a concentration comparable to that used in other studies of siRNA mediated gene silencing [20], while lower doses were found ineffective. The use of Alexa fluor 488 end labelled *myc*-siRNA enabled us to track *in vivo*, by fluorescence microscopy, the capability of naked siRNAs to cross *Hydra* cell membranes at pH 4 (Figure 1A–1B), with a greater efficacy compared to pH 7 (Figure 1C–1D), indicating that the effective delivery of the siRNA *in vivo* was enhanced by the acidic condition. Analysis of single cell suspensions shows delivery of labelled *myc*-siRNA to both epitheliomuscular and interstitial cells (Figure 1H–1M), while confocal microscopy analysis confirmed the localization within nests of nematoblasts (Figure 1E–1G), where *Hymyc1* is expressed. The silencing effect was evaluated at molecular level by quantitative Real Time PCR (qRT-PCR). Treatment with *myc*-siRNA for two days (2 d) caused a significant reduction of *Hymyc1* transcript levels at pH 4, but not at pH 7 (Figure 2A) suggesting that enhanced acidic-mediated uptake of RNA duplexes is required to induce specific gene silencing. The post-transcriptional silencing induced by *myc*-siRNA was confirmed at protein level by Western Blot analysis. Following preliminary semi-quantitative RT-PCR analysis (Figure 2B, upper panel), polyclonal antibodies raised in rabbit against HyMYC1 recombinant protein detected a drastic decrease of HyMYC1 protein in *myc*-siRNA treated animals by 2 d, compared to endogenous levels of *Hydra* actin (Figure 2B, lower panel). The specificity of the RNAi approach was assessed by testing unrelated siRNAs, such as *Luciferase* siRNA (*luc*-siRNA), designed on the firefly luciferase *GL2* gene. Results showed that *Hymyc1* transcription and translation were unaffected in *luc-*siRNA treated animals (Figure 2B). By qRT-PCR the potential adverse effects played by the acidic pH on gene expression were also analysed. As it might be argued that the acidic condition could play adverse effects on gene expression, we analysed the endogenous *Hymyc1* expression level in animals incubated at pH 4 for increasing periods, and compared them to expression levels in physiological condition (pH 7). The graph of Figure S3-A (Supporting Information) shows similar levels of *Hymyc1* transcripts both at pH 4 and pH 7, indicating that *Hymyc1* endogenous expression was unaffected by the acidic environment. Morphological analysis of treated animals further confirmed the absence of adverse effects played by the low pH on polyp viability (Figure S3-B of Supporting Information).

The efficiency of RNAi as function of the duration of treatment is shown in Figure 2C. Specific gene inhibition was detected in *myc*-siRNA treated animals by 2 d of treatment, when a 60% decrease in the *Hymyc1* mRNA amount was measured. Increasing the period of treatment up to 9 d did not further increase the silencing efficiency, probably due to fast cell turnover of the interstitial stem cells, continuously replacing the interfered cells or to the animal adaptation to the treatment. The long lasting effect of the RNAi was then investigated by performing a 4 d RNAi treatment followed by RNAi suspension and culturing in physiological condition, under normal feeding regime, over different periods. The graph of Figure 2D shows that *Hydra* polyps could recover from the RNAi treatment, restoring physiological levels of *Hymyc1* transcripts over the following five days. Thus the observed downregulation depends upon the continuous presence of siRNA oligonucleotides and indicates the reversibility of the siRNA mediated RNAi, similarly to other invertebrates RNAi knockdown models [21].

The efficiency of the new developed RNAi approach was tested on an additional gene, *β-catenin* (*β-cat*), a key gene involved in setting up the head organizer in *Hydra*. *β-cat* is an armadillo repeat-containing protein expressed weakly and uniformly throughout the polyp and at higher level in developing buds [22]–[23]. *Hydra* polyps treated for 2 d with specifically designed *β-cat*-siRNA revealed a strong and significant reduction (more than 50%) of the target mRNA (Figure S4), confirming the validity and the reproducibility of our RNAi strategy.

### *Hymyc1* gene silencing promotes proliferation of single and pairs of interstitial stem cells

**Figure 3:**
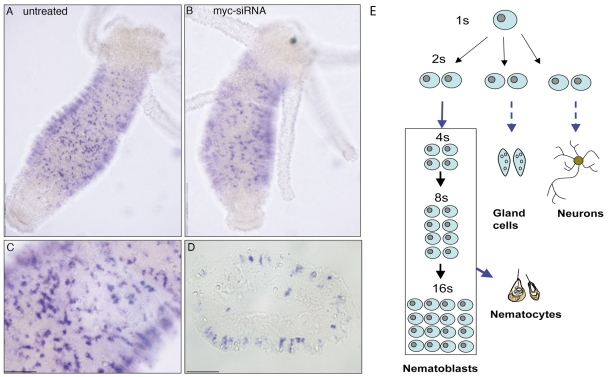
*Hymyc1* expression pattern is not affected by *Hymyc1* downregulation. A comparison of whole mount *in situ* hybridization performed on A) healthy animals and B) *myc*-siRNA treated animals (2 d) shows residual *Hymyc1* transcripts in the RNAi targeted cells. As previously reported [10] *Hymyc1* is expressed in proliferating interstitial cells, distributed along the gastric region, shown at higher magnification in (C). A cross section of stained animals confirms this expression pattern (D). Scale bars: 1 mm in A and B; 200 µm in C and D. A scheme of the differentiation pathways in the interstitial stem cell system is shown in (E). Interstitial stem cells include multipotent stem cells (1 s) and committed stem cells (2 s) that differentiate three major cell types in *Hydra* (nematoblasts, secretory cells, neurons, and gametes). Nematocytes, the phylum representative cells arming the tentacles and used for pray capture, originate from nematoblasts, i.e. committed cells that after several mitotic divisions (generating nests of 4, 8 and 16 cells connected by cytoplasmic bridges) finally differentiates into several types of nematocytes (stenoteles, desmonemes, isorriza, depending on the nematocyst morphology). 2 s cells committed toward either nematocyte, nerve or secretory cells, are morphologically indistinguishable. While single interstitial stem cells (1 s) continuously self renew, among the daughter cells (2 s) 60% remain stem cells, whereas 40% become committed to different cell types.

In order to detect differences in the expression pattern of *myc*-siRNA interfered animals whole mount *in situ* hybridizations using a Digoxigenin-labelled *Hymyc1* RNA probe were performed. Despite the decreased *Hymyc1* expression detected by qRT-PCR, differences in the signal distribution pattern between healthy and interfered animals (analysed at 2 d, 4 d and 9 d of continuous siRNA treatment) could not be appreciated, due to the Digoxigenin detection technique, not allowing quantitative measurements. As shown by stained specimens both whole mounted and cryo-sectioned (Figure 3A–3D) *Hymyc1* residual transcripts were detected in *myc*-siRNA treated animals in single and pairs of interstitial cells (1 s, 2 s), nests of nematoblasts (4 s−16 s), accordingly to previous reports [10]. The same expression pattern was detected in *luc*-siRNA treated animals, confirming the absence of aspecific effects due to the siRNA treatment (data not shown). A quantitative evaluation of the *Hymyc1* positive cells confirmed the absence of significant differences between interfered (by *myc*-siRNA and *luc*-siRNA) and control animals (Figure S5). This result suggests that the activation of the RNAi machinery occurred throughout the targeted cells, rather than on limited patches of cells where the siRNA could be selectively delivered.

Since the multipotent interstitial cells, their differentiation intermediates, and their product cells are all closely related (Figure 3E), changes in their relative population sizes may yield clues as to how these populations are controlled. A morphological analysis both at cellular level and in intact animals was then performed to investigate potential effects induced by *Hymyc1* silencing on the cell proliferation activity and cell type relative distribution.

**Figure 4:**
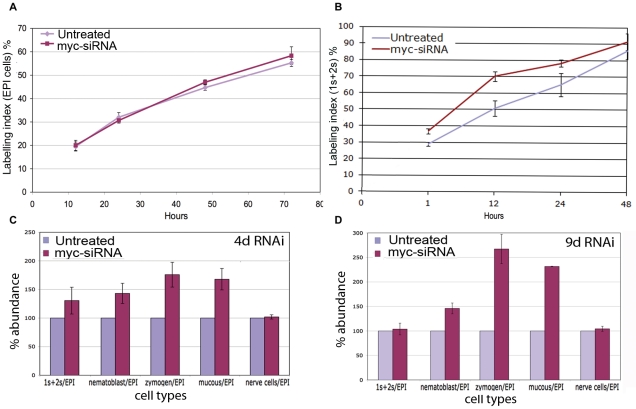
Effect of *myc* RNAi on cell cycling activity and cell type distribution. Cell cycling activity of epithelial cells (A) and 1 s+2 s stem cells (B) were obtained in control and treated animals by continuous incubation with BrdU, followed by maceration of ten animals at the indicated time points and fluorescence immunostaining. Data represent the average of three different experiments. In (C) and (D) the distribution of different cell types was assayed after 4 d and 9 d of *myc* RNAi, respectively. Five test animals were randomly selected from a pool of twenty five treated animals, and macerated for cell counting. The data represent the average of five independent RNAi experiments. Bars indicate standard errors. 1 s+2 s = single and pairs of interstitial cells; nematoblasts = nests of 4 s−16 s proliferating and differentiating nematoblasts; EPI = epithelial cells.

As *Hydra* epithelial cells proliferate with a cell cycle of about 3,5 d [24]–[25] and interstitial cells much faster with a cell cycle of about 1 d [26], the cycling activity of the epithelial and interstitial cells was assayed by continuous BrdU-labeling, over 3 and 2 d, respectively. Figure 4A shows that the proliferation rate of epithelial cells was not affected by *myc* RNAi, thus this cell type could be used to normalize relative cell type distribution. By contrast, proliferation of single and pairs of interstitial cells (1 s+2 s) was enhanced in myc-RNAi animals, indicating an effect of *myc* downregulation on the stem cell cycling activity (Figure 4B). The estimation of cell proliferation rate by mean of BrdU was carried out also in differentiating nematoblasts (4 s) and gland cells. Results reported in Figure S6 showed that *Hymyc1* RNAi does not affect proliferation rate of these cell types, suggesting a *Hymyc1* role played specifically on 1 s+2 s cells.

To gain insight into the possible effects of *Hymyc1* RNAi on the cell cycle, mitotic index and DNA content of 1 s+2 s cells were assessed. Counts of DAPI-stained cells by phase contrast microscopy combined with nuclear DNA staining revealed that the fractions of cells undergoing mitosis in myc-siRNA treated animals were 3,7% and 3,4% upon 3 and 4 d of treatment, respectively (n = 1000, for each treatment). Conversely, these fractions in untreated animals were 2,7% and 2,3% after 3 d and 4 d, respectively, indicating an increase of more than 40% of the mitotic index of 1 s+2 s cells as effect of RNAi. To check whether or not *Hymyc1* silencing controls specific cell cycle checkpoints, the DNA content of 1 s+2 s cells from RNAi animals was determined. We found not significant differences in the nuclear DNA profile between untreated and *Hymyc1* RNAi animals, suggesting that *Hymyc1* does not act as a regulator of one specific cell cycle checkpoint (Figure S7 of Supporting Information).

**Figure 5:**
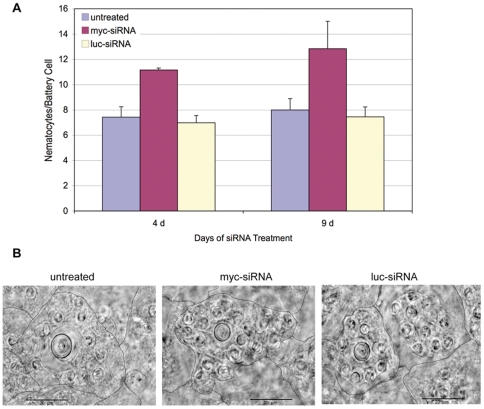
*Hymyc1* RNAi induces an increase in the nematocyte content of battery cells. (A) At time 4 d and 9 d of treatment with the indicated siRNA, animals were fixed and the number of nematocytes embedded in the battery cells, observed under the same focus plane, was scored. Data represent the average of measures from ten animals ± standard deviation. (B) Representative battery cells imaged from normal (left), *myc*-siRNA (middle) and *luc*-siRNA (right) treated animals, on whole mounts. Hand drawn black lines indicate the battery cell membranes. *Hymyc1* downregulation induces nematocyte differentiation and accumulation into battery cells. Scale bar: 20 µm.

To investigate the effects of *Hymyc1* downregulation on all interstitial cell lineage, cell type distribution was analysed in macerates at two time points, 4 d and 9 d, and the cells counted relative to epithelial cells, as not affected by *Hymyc1* RNAi (Figure 4A). After 4 d of siRNA treatment, the observed increased ratio 1 s+2 s/EPI was consistent with the boost of mitotic index and BrdU labelling previously reported (see above text and Figure 4B). Furthermore, we found in *myc*-siRNA treated animals an increase in the population of nematoblasts (4 s−16 s), zymogen and mucous cells, more pronounced at 9 d (Figure 4C, 4D), while nerve cells were not affected, suggesting a *Hymyc1* specific role in nematocyte and gland cell differentiation pathways. The population of mature nematocytes embedded in the battery cells was analysed in tentacles of intact fixed animals. *Hydra* tentacles are structured as cell bilayers, as the rest of the animal body, but contain specialized ectodermal cells, the battery cells (Figure S1) [27]–[28]. These tentacle specific cells arise from terminal differentiation of ectodermal cells, including the mitosis arrest and the inclusion into the cytoplasm of neuronal cells and several nematocytes, the stinging cells involved into the prey capture. This complex system, the battery cell, is recognizable by phase contrast optical microscopy. Results of Figure 5A show that *myc*-siRNA interfered animals, both at 4 d and 9 d, presented an increased ratio of mature nematocytes/battery cells, compared to relative control sets of animals and a representative battery cell is reported in Figure 5B. This effect was induced by *myc*-siRNA treatment in almost all individuals, as shown by the distribution of the ratios among ten animals (Figure S8).

**Figure 6:**
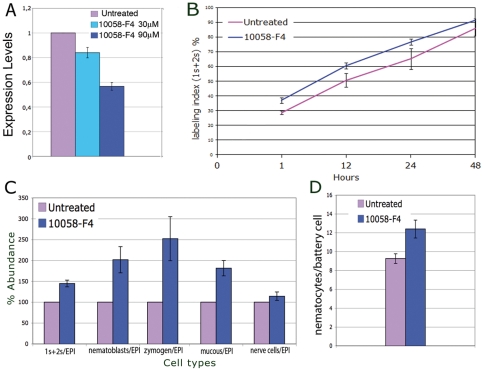
Molecular and morphological alteration induced by the c-myc inhibitor 10058-F4. A) Living polyps were treated with the *c-myc* inhibitor 10058-F4 for 48 h at two different concentrations (30 µM and 90 µM) before RNA extraction for qRT-PCR analysis. Expression levels, relative to *HyEF1*α, indicate *Hymyc1* silencing in a dose dependent manner, reaching 47% downregulation at the higher dose tested. Error bars indicate standard deviations calculated from three independent experiments, each performed in triplicate. B) Cell cycling activity of 1 s+2 s stem cells were obtained in control and treated animals (as above, 10058-F4 90 µM) by continuous incubation with BrdU, followed by maceration of ten animals at the indicated time points and fluorescence immunostaining. Labelling indexes of 4 s and gland cells are reported in Figure S6 of Supporting Information. C) Single cell suspensions were prepared from polyps treated as in A and the relative cell types counted by phase contrast microscopy. Treatment with 10058-F4 enhances stem cell proliferation and determinates the accumulation of intermediate and terminal differentiation products. D) Polyps treated with the *c-myc* inhibitor as in A were fixed and whole mounted for analysis of the nematocyte content into the tentacles (as described in Figure 5). The small molecule myc inhibitor induces an increase in the nematocyte content of battery cells, identical to the effect induced by *myc*-siRNA.

The specificity of the phenotype induced by *myc*-siRNAi was tested by treating animals with a biochemical c-MYC inhibitor, the thioxothiazolidinone10058-F4 [29], which blocks c-MYC/MAX heterodimer formation through molecular mechanisms poorly understood so far, and it affects as well the *myc* gene transcription level [30]–[31]. Treatment of living polyps with 10058-F4 for 2 d induced 50% reduction of *Hymyc1* expression levels, as assayed by qRT-PCR (Figure 6A), while analysis of cell proliferation rate showed an effect on 1 s+2 s cells (Figure 6B), but not on other cell types (Figure S6 of Supporting Information). Cell type distribution analysis also revealed increased proliferation of 1 s+2 s stem cells, and increased amounts of intermediates and terminal differentiation products (nematoblasts, nematocytes, secretory cells) (Figure 6C). As detected in *Hymyc1* RNAi polyps, also the phenotype of the battery cells was affected, containing an abnormal number of nematocytes (Figure 6D), indicating unequivocally the specificity of the *Hymyc1* downregulation effects, and indirectly the structural and functional conservation of the HyMYC1/HyMAX complex. Taken together, these findings suggest that genetic and biochemical repression of *Hymyc1* activity affects in the same way the interstitial cell lineage homeostasis: *Hymyc1* conditional elimination enhanced stem cell proliferation, and in turn unbalances the differentiating products.

As *Hymyc1* is expressed in proliferating stem cells of the interstitial cell lineage, we also evaluated its expression profile during regeneration, a complex phenomena involving extensive cell reprogramming, proliferation and differentiation processes to rebuild a new organism from an amputated moiety [32].

**Figure 7:**
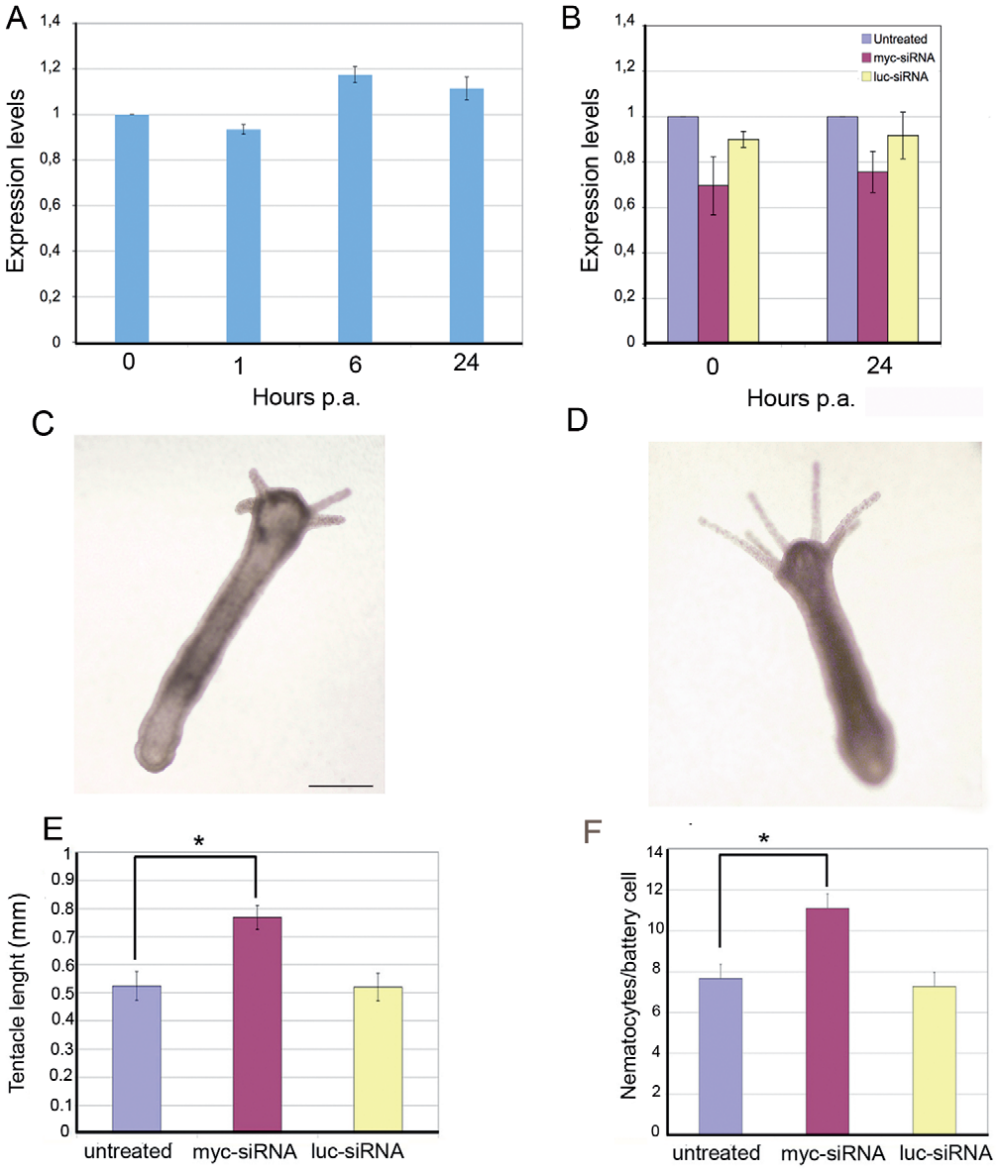
Effect of *Hymyc1* RNAi on regeneration. A) Healthy animals were bisected at time t = 0 and allowed to regenerate for the indicated period, expressed in hours. At different times post amputation (p.a.) total RNA was extracted and analysed by qRT-PCR. Not significant differences in *Hymyc1* expression levels were detected during the regenerative process. B) *myc*-siRNA treatment (4 d) caused a reduction of *myc* mRNA levels. At this point (time t = 0) animals were bisected and allowed to regenerate for the indicated hours. Downregulation effects persist during regeneration. Each error bar indicates standard deviation calculated from triplicates. C–D) *myc*-siRNA treated animal present abnormal tentacle morphogenesis. Representative phenotypes of head regeneration at 72 hr post amputation are shown. A *myc*-siRNA treated animal (D) presents tentacles of increased length compared to untreated (C) or *luc*-siRNA treated polyps (not shown). Scale bars: 500 µm. E) The average of tentacle length, calculated on a subset of 20 animals/condition, indicates the faster tentacle development induced by *myc-*RNAi. Asterisk indicates p<0,05, according to t-Student test). F) The average nematocyte content of the battery cells was scored on tentacles of regenerating animals at 96 hr post amputation. *myc*-RNAi promotes nematocyte differentiation.

Quantitative analysis of *Hymyc1* expression performed over the first 24 h post amputation indicates that *Hymyc1* expression levels were not specifically modulated during the early stages of the regenerative process of healthy animals (Figure 7A). When analysing *myc* RNAi regenerating animals, similar *Hymyc1* expression levels were found (Figure 7B) indicating that the RNAi effect was maintained during the regeneration and it could possibly affect cell type distributions. To this aim, the phenotype of the regenerating animals was analysed in more detail. *In situ* hybridization on *Hymyc1* interfered regenerating animals, similarly to what observed on intact animals, did not reveal quantitative differences in the *Hymyc1* expression pattern (data not shown), while a clear morphological phenotype was detected in the regenerated tentacles: the average tentacle length and nematocyte content per battery cells were significantly increased (Figure 7D–7F) indicating that *Hymyc1* RNAi effect persists throughout the regenerative process and affects tentacle morphogenesis. These results substantiate the view of a role of *Hymyc1* in the control of proliferation/differentiation rate in *Hydra*.

## Discussion

The possibility to perform gain- or loss- of function approaches to analyse gene function may add new values to the widespread use of *Hydra* as a genomic model organism with a pivotal position in the evolutionary tree. The generation of transgenic polyps by embryo microinjection is a well-established technique [33] and several methods have been successfully used to do RNAi in adults. The published RNAi methods rely on long dsRNA delivered through different techniques, spanning from whole animal electroporation [13] to localized electroporation [15], and used successfully for functional analysis of genes involved in head regeneration [15]–[34]–[35]. In order to reduce major side effects as tissue necrosis and lethality, a milder strategy for dsRNA delivery through feeding of dsRNA producing bacteria has been developed and used for silencing of both endodermal and ectodermal expressed genes [36]–[37], while the soaking method using long dsRNA has been employed for efficient RNAi of key developmental genes in the marine hydroid *Hydractinia echinata* [38]. We developed in this paper a novel approach of RNAi in *Hydra*, based on siRNA oligonucleotides. First discovered in plants and in the nematode *Caenorhabditis elegans* [16]–[39]–[40] the production of siRNAs that bind to and induce also systemically [41] the degradation of specific endogenous mRNAs is now widely employed to inhibit gene function and holds great potentials in the therapeutic treatment of several diseases [42]–[44]. At cellular level, duplex siRNAs are negatively charged polymers and so cannot easily penetrate hydrophobic cellular membranes without assisting carriers such as cationic polymers, liposomes or nanoparticles. We succeeded in our delivering method by working in acidic conditions, which resulted in siRNA charge reversal and great enhancement of siRNA intracellular delivery. Alexa 488 labelled *myc*-siRNAs were found into the cytoplasm of *Hydra* ectodermal cells, within both epitheliomuscular and interstitial cells, as granular structures with different sizes, not excluding the presence of single units, undetectable by confocal microscopy analysis (Figure 1). The experimental conditions used for siRNA delivery confirm previous data on the importance of the positive surface charge of delivery vehicles in promoting cell uptake [12]–[45]–[46]. In *Drosophila* an active and specific pathway that involves clathrin-mediated endocytosis has been found responsible for siRNA uptake [47] and accumulation in vesicles, while the mechanism of cytoplasmic release to enter the RNAi machinery has not been identified to date. Although it might be argued that by our approach siRNA delivery occurs more into ectodermal cells rather than into interstitial cells, the strong molecular downregulation observed suggests that the RNAi response results from both direct delivery to interstitial cells and spreading effect of the RNAi response, similarly to other invertebrates [41]–[47]. This hypothesis is supported by the presence in the *Hydra* genome of predicted genes encoding for components required for amplification and systemic spread of an RNAi signal, i.e. RNA-dependent RNA polymerase (RdRP) and SID proteins (Hma1.117083 and Hma1.114101 respectively). Moreover, the cytoplasmic bridges present among nematoblasts might provide direct passage of siRNA signals through connected cells to enable RNAi. We targeted our RNAi approach to *Hymyc1* gene and showed the specificity of our method including luciferase- and β-catenin- siRNA as negative and positive controls, respectively. While β-cat silencing proved the robustness and reproducibility of our RNAi method, *luc*-siRNA ineffectiveness in *Hymyc1* downregulation demonstrated the absence of harmful sequence independent side effects elicited by the siRNA treatment: phenotypical, cellular and molecular analysis showed that *luc*-siRNA did not alter *Hymyc1* expression, nor interfered with proliferative/differentiating processes leading to abnormal phenotypes.

**Figure 8:**
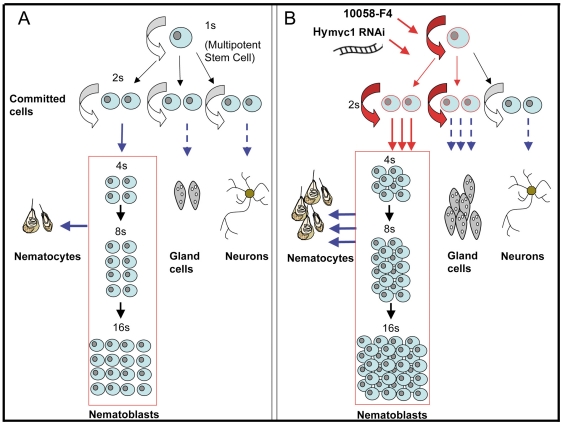
Interstitial cell differentiation pathways. Schematic representation of the multiple differentiation pathways of the interstitial stem cells in homeostatic condition (A) and the effects on stem cell proliferation/differentiation induced by genetic or biochemical *Hymyc1* downregulation (B). *myc*-siRNA treatment or *myc* biochemical inactivation induce a moderate increase in the 1 s and 2 s stem cell self-renewal and proliferation activity (red arrows). This in turn generates a higher number of both differentiating intermediates (nematoblasts) and terminal differentiated products such as nematocytes and gland cells (blue arrows). Dashed arrows indicate the absence of morphological distinct cellular intermediates. *Hymyc1* downregulation does not affect the nerve differentiation pathway, consistent with the absence of *Hymyc1* expression in this cell type.

*Hymyc1* is homologue of the c-*myc* protooncogene, a key gene conserved throughout the animal kingdom [48], controlling several fundamental processes of the cell cycle, both intracellular functions (cell growth, proliferation, apoptosis) and extracellular processes that coordinate cell proliferation with the microenvironment (the stem cell niche) [49]. Although the molecular, biochemical and functional analyses of *Hymyc1* has highlighted structural conservation in the featuring domains (i.e. bHLH-Zip domain and *myc*-boxes I to III) and functional similarities to the vertebrate derivatives [10], its physiological role in *Hydra* has not been elucidated so far. By challenging living *Hydra* with *myc*-siRNAs, we specifically downregulated *Hymyc1* mRNA and protein levels. The inhibition was achieved after 2 d of treatment, while continuous incubation up to 9 d, under normal feeding regime, did not cause cumulative effects, probably due to saturation of the RNAi machinery, or to other mechanisms of animal adaptation. Our experimental conditions ensured animal health all along the treatment period and the absence of stressing condition (such as starvation or chemical/physical compounds required for efficient delivery of nucleic acids), which can complicate the interpretation of the phenotypical effect of gene silencing. Despite the presence of an additional *myc* orthologue gene in the *Hydra* genome database (*Hymyc2*) which could possibly interfere with *Hymyc1* downregulation, a clear phenotypical effect was detected in *myc*-siRNA treated animals. At cellular level, an extensive analysis of single cells prepared by maceration revealed abnormal distribution ratios between cellular types of the interstitial cell lineage. As the density of the stem cell population (i.e 1 s+2 s/EPI) is species-specific and dependent upon environmental stimuli, such as the feeding regime [6]–[25]–[26], we did not report the absolute values, but pointed out to their changes relatively to control animals. While not affecting the epithelial cell proliferation rate, *Hymyc1* downregulation induced a moderate increase in the stem cell proliferation rate and density, which produced dramatic effects on the related cell population sizes. These results are in line with previous studies relating stem cell self-renewal to the cell density, and showing that nematocyte commitment increases as stem cell concentration increases [[5]–[24]–[26]. The abnormal numbers of mature nematocytes embedded in the tentacle battery cells confirmed the burst in the nematocyte differentiation. Although we cannot exclude an effect on nerve cell commitment, the measure of mature nerve cells, found unaffected by *Hymyc1* repression, ruled out an enhancement in nerve cell differentiation. By contrast, the secretory cell differentiation pathway was profoundly activated, as shown by the doubled numbers of both zymogen and mucous cells. Interestingly, at 9 d of treatment, the stem cell density was restored to physiological levels, suggesting a negative feedback between stem cells, as elsewhere proposed [6]. The specificity of the effects produced by *Hymyc1* inhibition was confirmed using a chemical inhibitor of the MYC/MAX interaction, 10058-F4 [29]. Treatment of living polyps with this inhibitor induced *Hymyc1* downregulation, as well as increased 1 s+2 s stem cell proliferation and enhanced differentiation of nematoblasts, secretory cells and nematocytes. A schematic model of the effects of both the genetic and biochemical *Hymyc1* inhibition on the proliferation/differentiation rates is shown in Figure 8. Altogether these results suggest a role of *Hymyc1* in the negative regulation of the stem cells where it is expressed: the decreased expression enhances stem cell proliferation and in turn the differentiation of intermediates and terminal derivatives. The increase in stem cell density may finally inhibit the stem cell self-renewal capability over long periods.

Of intriguing interest is the effect displayed by *Hymyc1* RNAi on *Hydra* regeneration. qRT-PCR showed that, 24 h post amputation *Hymyc1* transcription levels are similar to pre- amputation. Regenerated heads also presented morphological traits similar to the pre-amputated animals, i.e longer tentacles armed with an abnormal number of nematocytes, suggesting the persistence of the RNAi response. Consistent with this hypothesis are evidences from *C.elegans*, in which effective interference is observed in the progeny of dsRNA injected animals at early larval stages [21], and from the planarian flatworm *Schmidtea mediterranea*, where gene inhibition persists throughout the process of regeneration [50].

The involvement of c-*myc* in controlling cell proliferation and differentiation processes has been shown in several systems, by both gain and loss of function approaches. In murine hematopoietic stem cells (HSC) the group of Trumpp showed that c-*myc* conditional elimination resulted in the accumulation of HSCs *in situ* due to their failure to initiate normal stem cell differentiation [51]–[52]. In human pancreatic endocrine cell lines *c*-*myc* downregulation by RNAi induces cells to exit the cell cycle and enter the differentiation pathway, thus c-*myc* plays a role in the switch mechanism that controls the inverse relationship between proliferation and differentiation [53]. In *Drosophila* mutation of the *Drosophila myc* gene results in small flies due not to fewer cells but to their cells which are smaller in size [54], suggesting that *dmyc* participates in the regulation of cell mass. Although *myc* and the members of the *myc* “network” are encoded in the genomes of most metazoan phyla and many aspects of its biology may arise from comparative analysis, the disparate cellular events seeing its involvement make it difficult to evaluate *myc*'s function in its entirety due to redundancy among family members, tissue specificity, and complex phenotypes.

In Cnidaria, the molecular mechanism underlying RNAi have not been elucidated so far. We show the capability of siRNA to enter *Hydra* cells and to trigger a RNAi response, similarly to many metazoans. Many other aspects of the *Hydra* RNAi response need to be addressed now, from the systemic long range character of the RNAi response, to the mechanisms underlying the uptake of siRNA, to the deviation from standard endocytic uptake at some point to deliver dsRNA to the cytoplasm, to date unknown in any model of RNAi. The current design in our group of nanocarriers to improve siRNA delivery, self-tracking and specificity will surely provide in the near future new tools to decipher the mechanisms of RNA interference in *Hydra*.

In conclusion, this study focuses on *Hymyc1* silencing, through an innovative RNAi strategy enhancing siRNA uptake in the polyp, and describes how *Hymyc1* knockdown affects the developmental dynamics of stem and interstitial cell lineage in *Hydra*. In particular, our results add significant value to fundamental studies on the stem cell biology addressing the mechanisms by which these cells maintain the balance between self-renewal and differentiation. As *myc* deregulated expression occurs in the majority of human cancers, understanding the ancient roots of *myc* biology may be of interest for the wide scientific community targeting *c-myc* for therapeutic purposes. For scientists working on cnidarians this paper opens new avenues to decipher the molecular control of the cellular plasticity underlying growth and proliferation in *Hydra*.

Finally, the establishment of a RNAi method which avoids invasive procedures while ensures effective delivery of siRNA under normal culturing condition, lays the foundations of a comprehensive analysis of the RNAi response in *Hydra* allowing to track back in the evolution the origin of the RNAi response.

## Materials and Methods

### Culture of animals and regeneration experiments

*Hydra vulgaris* (strain Zurich, originally obtained by P.Tardent) were asexually cultured in *Hydra* medium (1 mM CaCl_2_, 0.1 mM NaHCO_3_, pH 7) by the method of Loomis and Lenhoff with minor modifications. The animals were kept at 18±1°C and fed three times per week with freshly hatched *Artemia salina* nauplii. Polyps from homogeneous populations, three-weeks-old and carrying one or two buds, were selected for the experiments. For regeneration experiments, treated polyps (4 d of treatment) were bisected in the upper gastric region and monitored at various time points post amputation.

### *In vivo* RNA interference through small interfering RNAs

For RNA interference groups of 25 animals were collected in plastic multiwells, allowed to equilibrate at room temperature in 1 ml of *Hydra* medium at pH4. The test was initiated by adding 70 nM siRNA to each well containing the animals, since then continuously exposed to the siRNA oligonucleotides, under normal feeding regime. After the washing procedures new siRNA oligonucleotides were supplied. 21 bp long siRNA targeting *Hymyc1* were designed using on line siRNA design services and purchased by QIAGEN. Both unlabelled and 3′- Alexa fluor 488 siRNA were designed on the *Hymyc1* DNA target sequence: 5′- AAGATGCTCACGCGTCAAGAA-3′. As control of silencing specificity, siRNA oligonucleotides targeting the Luciferase GL2 gene were purchased by QIAGEN (cat no 1022070). Moreover, as positive control to assess the reliability of this RNAi method, β-catenin gene expression was interfered by using a specific 21 bp long siRNA corresponding to nucleotides 750–770 of *Hydra vulgaris* β-catenin mRNA (GenBank Accession no. U38624.1). The *myc* inhibitor 10058-F4 (5-[(4-Ethylphenyl)methylene]-2-thioxo-4-thiazolidinone) was purchased by Sigma-Aldrich (cat no. F3680). Groups of 25 animals were incubated for 48 h in presence of 10058-F4 (30 µM or 90 µM), and then used for molecular and cellular characterization, as described in the following sections.

### Imaging

siRNA uptake was monitored *in vivo*, using a Camedia-digital camera (Olympus) connected to a stereomicroscope (Olympus ZSX-RFL2) equipped with fluorescence filter sets (BP460- 490/DM505/LP510). Following extensive washes, *in vivo* imaging was accomplished by an inverted microscope (Axiovert 100, Ziess) equipped with a digital colour camera (Olympus, DP70) and fluorescence filter sets (BP450-490/FT510/LP515). For imaging acquisition and analysis the software system Cell F (Olympus) was used. Confocal images were collected with a Leica TCS SP2 AOBS confocal microscope (Mannheim, Germany) with 40× oil immersion optics. Laser lines at 488 nm and 633 nm for excitation of Alexa fluor 488 and TOPO-ETC were provided by an Ar laser and a HeNe laser, respectively. Detection ranges were set to eliminate crosstalk between fluorophores. Tissue imaging of interfered animals was performed under bright field.

Quantification of *in situ* hybridization signal for *Hymyc1* was accomplished as it follows: whole mounted animals were imaged by an inverted microscope (Axiovert 100, Ziess) equipped with a digital colour camera (Olympus, DP70). Images were taken under the same conditions of acquisition (light and exposure time), saved in tagged-image file format (TIFF) at a size of 1300×1030 pixels, posterized by Adobe Photoshop by setting the same threshold levels and then converted in HSB format for data processing using the Image processing and Analysis software Image J (Version 1.45i).

### Gene and protein expression analyses

Whole-mount *in situ* hybridization using DIG-labelled RNA probe was carried out as described previously [55] using NBT/BCIP (Roche) as substrate for staining. The *myc* riboprobe was synthesized using the plasmid *Hymyc1* (GenBank Accession no. GQ856263) as template and used at a concentration of 0.1 ng/ml for hybridization. The *Hymyc1* plasmid was produced as described [10].

Total RNAs from treated and untreated animals was purified using Tri Reagent (Molecular Research Center) and its concentration was quantified on the NanoDrop ND-1000 spectrophotometer (Thermo Scientific, USA). The first-strand cDNA synthesis was carried out with the SuperScript II Retrotranscriptase (Invitrogen) and oligo dTs, using 0.5 µg of DNA-free RNA in a final volume of 25 µl, according to the manufacturer's instructions. Semiquantitative RT-PCR was employed to estimate myc mRNA levels. Each PCR reaction was carried out with Platinum Taq DNA Polymerase kit (Invitrogen) in a total volume of 25 µl containing 1 µl cDNA and 0.5 µM primers for *Hymyc1* (forward: AGGACGAAGTTGATGTAGTTGGA; reverse: 5′-GCGAAGCAACTTTCAGTATTGTTAC-3′ and *Hydra* Elongation factor 1α (5′-ATGATTGAACCATCCCCAAA-3′; 5′GCTTCAATGGCAGGATCATT 3′) as reference gene. After initial denaturating step of 5′ at 94°C, cycling steps were as follows: 30 s at 94°C, 30 s 58°C and 30 s at 72°C. For comparative analysis, the experiments were done in triplicate and data were collected at different end point (25, 30 and 35 cycles).

Quantitative Real-Time polymerase chain reaction (qRT-PCR) was performed in 25 µl of reaction mixture consisting of 1× Platinum SYBR green qPCR SuperMix-UDG with ROX (Invitrogen), serial cDNA dilutions and 0.5 µM each primer. The reactions were processed using the StepOne Real-Time PCR system (Applied Biosystem) under the following cycling steps: initial denaturation for 10 min at 94°C, followed by 40 cycles at 94°C for 15 s, 58°C for 30 s, and 72°C for 30 s, and a 20 min gradient from 55°C to 90°C to obtain a melting curve. To normalize RNA levels, *Hydra* Elongation factor 1α gene (HyEf-1α GenBank Accession no. Z68181.1) was employed as internal calibrator. Gene-specific primers (HyEf-1α: forward, 5′CCAGGAGACAATGTCGGTTT 3′; reverse, 5′GCTTCAATGGCAGGATCATT 3′; Hymyc1: forward, 5′ TACAGAAAGCGAGGACGAAGTT 3′; reverse, 5′GCGAAGCAACTTTCAGTATTGTTAC 3′; β-cat: forward, 5′GGCGCTCTTCACATTTTAGC 3′; reverse, 5′TGCACCTTCACGCTCAATAG 3′) were designed using Primer3 software (http://frodo.wi.mit.edu/primer3/); the lengths of *HyEf-*1α and *Hymyc1* amplicons were 100 bp and 157 bp, respectively. At least three independent repeats for each experiment were carried out. Herein, the delta–delta Ct (2^−ΔΔCT^) method, for comparing relative expression results between treatments, was applied [56].

For Western blot analysis total proteins were extracted from groups of 25 animals, using the triple detergent buffer according to Sambrook *et al*. [57]. The resulting homogenate was centrifuged for 15 min at 14000 *g* and the proteins contained into the supernatants collected and quantified by the Bradford method using BioRad reagent with BSA as a standard. Equal amounts of proteins were separated on 12% SDS-polyacrylamide gel and transferred onto nylon membranes (BioRad, San Diego, CA). MYC protein levels were detected using anti-HyMYC1 antibody (1∶500, kindly provided by K.Bister, University of Innsbruck) and compared to actin proteins, using an anti-actin primary antibody (1∶100, Sigma) to probe an identical blotted gel.

### *Hydra* cell and tissue analysis

Analysis of siRNA treated animals was done both on whole animals and isolated fixed cells. Animals were anaesthetized in 2% urethane in *Hydra* medium for 2 min. The relaxed and elongated *Hydra* were fixed with Lavdowsky's fixative (ethanol: formalin: acetic acid: water-50∶10∶4∶40), rehydrated, and mounted on microscope slides in Glycerol 50% in PBS (8 g/l NaCl; 0.2 g/l KCl; 1.44 g/l Na_2_HPO_4_.7H_2_0; 0.24 g/l KH_2_PO_4_). The tentacles were examined for two types of nematocyst capsules (stenoteles and desmonemes) under an optical microscope. The battery cell structure, indeed, makes it possible to count the nematocytes with phase optics (32×) on the surface of a fixed tentacle facing the objective. Each tentacle was divided into three ideal sections of different length from the base to tip, and the number of nematocytes present in the middle section was counted. The ratio nematocyte/battery cell was calculated on a total of one hundred battery cells, collected on ten different polyps. An estimate of the tentacle length during head regeneration was calculated on images from both untreated and siRNA treated animals using the Cell F software (Olympus). At least 25 animals/condition were analysed.

For analysis of cell type distribution whole animals were macerated into a suspension of fixed single cells as described [58]. Five animals per condition were macerated at the indicated time of treatment and scored for single and pairs of interstitial cells (1 s+2 s), nests of four –sixteen proliferating or differentiating nematoblasts (4 s−16 s), zymogen and mucous cells, epithelial cells (EPI). An average of 300 EPI cells was counted, per condition. For cell nuclei detection cells were stained with 4′,6-diamidino-2-phenylindole dihydrochloride (DAPI) 0,25 mg/L before mounting. For analysis of proliferation rates, intact *Hydra*, untreated or treated for 48 hr with *myc*-siRNA, were continuously incubated with bromodeoxyuridine (BrdU) 5 mmol/l (Sigma) for 1, 12, 24, 48 and 72 hr, immediately fixed, and macerated cells spread on microscope slides as above described. BrdU incorporation in proliferating cells was detected by immunolocalization using mouse anti BrdU monoclonal antibody (1∶500, Sigma), and Novolink Polymer detection System (Novocastra Laboratories ltd.). To further estimate cell proliferation, mitotic index was measured on macerates through phase contrast optical microscopy combined with Hoechst 33342 staining of nuclear DNA by counting the percentage of cells undergoing mitosis (n = 1000 cells for each treatment). To quantify cell cycle distribution, DNA content profiles were determined on Hoechst 33342-stained macerates. Fluorescence intensity of individual nuclei was quantified by using the Fiji program from ImageJ software package.

For tissue sectioning, test animals were soaked over night in 30% sucrose in PBS and then embedded in the frozen section medium Neg-50 (Richard-Allan Scientific). Cryo-sections of 10 µm thickness were obtained by a cryostat (Leitz, digital 1760), collected on gelatine coated slides (Superfrost microscope slides, Menzel) and mounted in D.P.X (Sigma) before imaging.

### Statistical analysis

Tentacle length measurements, number of nematocyte per battery cell, relative cell type counting and qRT-PCR results were expressed as means±SD. The statistical significance of the results was determined by analysis of Student's *t* test. *p* values of <0.01 were considered highly statistically significant (two asterisks). *p* values of <0.05 were considered statistically significant (one asterisk).

## Supporting Information

Figure S1 **Structural anatomy of** ***Hydra vulgaris*** **.** a) Picture of living *Hydra*. The animal has a simple body plan: it is a tube with a head at the apical end, and a foot, or basal disc at the other. The head is in two parts, the hypostome (mouth) at the apex, and below that the tentacle zone from which a ring of tentacles emerge. Scale bar 200 µm b) the bilayered structure of the animal: the body wall is composed of two self renewing cell layers, an outer ectoderm and an inner endoderm, separated by an extracellular matrix, the mesoglea. The arrows on the left side indicate the direction of tissue displacement c) longitudinal sections at level of tentacle (upper figure) and gastric region (lower figure). On the tentacle, the ectodermal cells (ec) are called battery cells and contain embedded several types of nematocytes (nem), one sensory neuron facing the exterior (sn), a ganglial neuron (gn) making connections both with other cells and to myonemes. Along the animal body both ectoderm and endoderm layers are composed of epitheliomuscular cells, while interstitial stem cells and their intermediate and terminal derivatives (neurons, nematocytes and secretory cells) are interspersed among ectoderm and endoderm.
(TIF)

Figure S2 **Zeta potential of siRNA as a function of pH.** The zeta potential of siRNA depends on the pH of the solution: while at neutral pH siRNA show negative zeta potential values due to the presence of phosphate groups of the RNA backbone, at acidic pH, in *Hydra* culture medium, the presence of calcium divalent ions neutralize the negative charges, conferring a positive net charge to the molecules. The positive charge influences the electrokinetics of the siRNA molecules, promoting their intracellular uptake. Measurements were performed in Hydra medium, at the indicated pH.
(EPS)

Figure S3 **Acidic pH does not affect** ***Hymyc1*** **expression levels.** A) Endogenous *Hymyc1* expression levels were measured by qRT-PCR on animals kept for the indicated periods at neutral or acidic pH. Not significant differences were induced by the different conditions. Data are mean of three independent experiments. B) Image of a representative polyp incubated at in *Hydra* medium at pH 4 for 4 days. The polyp appears healthy, with long extended tentacles and presents no signs of toxicity induced by the treatment.
(EPS)

Figure S4 **Efficient silencing of β-catenin gene through siRNA mediated RNAi.** qRT-PCR was performed on total RNA extracted from polyps treated 2 d with *Hydra* β-catenin specific siRNA. A significant reduction of the Hyβ-cat transcript levels (60%), compared to *HyEF1*α, was induced by Hyβ-cat siRNA, showing the reliability and robustness of our approach. Two asterisks, p<0.01, according to t-Student test.
(TIF)

Figure S5 **Quantification of** ***in situ*** **hybridization signal for** ***Hymyc1*** **mRNA.** The Image processing and Analysis software Image J (Version 1.45i) was used to quantify the signal intensity produced by *in situ* hybridization in control, *myc*-siRNA and *luc*-siRNA treated animals, using *Hymyc1* as probe. Not significant differences were detected as effect of siRNA treatment, indicating residual *Hymyc1* transcripts in *myc*-RNAi animals. On the other side, *luc*-siRNA animals were not affected in *Hymyc1* expression, confirming the absence of putative side effects.
(TIF)

Figure S6 **Effect of** ***Hymyc1*** **RNAi on nematoblast and gland cell proliferation.** Cell cycling activity of A) nematoblasts (4 s) and B) gland cells. Control untreated animals (incubated at pH 4) and *myc*-siRNA treated animals were continuously incubated with BrdU (red line) and with the *c-myc* inhibitor 10058-F4 (90 µM, green line), before maceration at the indicated time points and fluorescence immunostaining. Data represent the average of three different experiments. Not significant differences were observed in the proliferation rates of 4 s and gland cells induced by *myc*-siRNA or 10058-F4 treatments.
(TIF)

Figure S7 **Nuclear DNA content of interstitial stem cells (1 s+2 s) in** ***myc-*** **siRNA treated** ***Hydra*** **.** A) Hoechst 33342 staining and B) phase contrast image of macerated cells. 1 s and 2 s: large interstitial stem cells (note that 1 s is in mitosis); ecto: ectodermal epithelial cell; endo: endodermal epithelial cell; gc: gland cell. C) After three or four days of siRNA treatment, the nuclear DNA profile of 1 s+2 s shows no significant differences as compared with untreated polyps. Nuclei of 25 nerve cells and differentiated nematocytes, which are terminally arrested in G1, were used to determine the fluorescence intensity of the 2C DNA content. Procedures: Polyps were treated for three or four days with *myc-* siRNA. Then, treated and untreated animals were macerated and spread onto microscope slides. After drying, the maceration preparations were stained with Hoechst 33342. Fluorescence intensity of individual nuclei was finally quantified by using the Fiji program of the ImageJ software package.
(TIF)

Figure S8 **Distribution of the ratio nematocyte/battery cells among different animals.** At time 4 d and 9 d of treatment with the indicated siRNA, animals were fixed and examined under an optical microscope. Under fixed focus plane, the ratio nematocyte/battery cell was calculated on a total of one hundred battery cells, collected on ten different polyps. *myc*-siRNA specifically induces an increase in the nematocytes embedded in the tentacle battery cells.
(TIF)

## References

[1] Askew DS, Ashmun RA, Simmons BC, Cleveland JL (1991). Constitutive c-myc expression in an IL-3-dependent myeloid cell line suppresses cell cycle arrest and accelerates apoptosis.. Oncogene.

[2] Bosch TC (2009). Hydra and the evolution of stem cells.. Bioessays.

[3] Bode HR (1996). The interstitial cell lineage of hydra: a stem cell system that arose early in evolution.. J Cell Sci.

[4] Watanabe H, Hoang VT, Mattner R, Holstein TW (2009). Immortality and the base of multicellular life: Lessons from cnidarian stem cells.. Semin Cell Dev Biol.

[5] Campbell RD, David CN (1974). Cell cycle kinetics and development of Hydra attenuata. II. Interstitial cells.. J Cell Sci.

[6] Holstein TW, David CN (1990). Cell cycle length, cell size, and proliferation rate in hydra stem cells.. Dev Biol.

[7] Amati B, Brooks MW, Levy N, Littlewood TD, Evan GI (1993). Oncogenic activity of the c-Myc protein requires dimerization with Max.. Cell.

[8] Evan GI, Littlewood TD (1993). The role of c-myc in cell growth.. Curr Opin Genet Dev.

[9] Shi Y, Glynn JM, Guilbert LJ, Cotter TG, Bissonnette RP (1992). Role for c-myc in activation-induced apoptotic cell death in T cell hybridomas.. Science.

[10] Hartl M, Mitterstiller AM, Valovka T, Breuker K, Hobmayer B (2010). Stem cell-specific activation of an ancestral myc protooncogene with conserved basic functions in the early metazoan Hydra.. Proc Natl Acad Sci U S A.

[11] Elbashir SM, Harborth J, Lendeckel W, Yalcin A, Weber K (2001). Duplexes of 21-nucleotide RNAs mediate RNA interference in cultured mammalian cells.. Nature.

[12] Tortiglione C, Quarta A, Malvindi MA, Tino A, Pellegrino T (2009). Fluorescent nanocrystals reveal regulated portals of entry into and between the cells of Hydra.. PLoS One.

[13] Lohmann JU, Endl I, Bosch TC (1999). Silencing of developmental genes in Hydra.. Dev Biol.

[14] Miljkovic M, Mazet F, Galliot B (2002). Cnidarian and bilaterian promoters can direct GFP expression in transfected hydra.. Dev Biol.

[15] Smith KM, Gee L, Bode HR (2000). HyAlx, an aristaless-related gene, is involved in tentacle formation in hydra.. Development.

[16] Elbashir SM, Lendeckel W, Tuschl T (2001). RNA interference is mediated by 21- and 22-nucleotide RNAs.. Genes Dev.

[17] Naito Y, Yoshimura J, Morishita S, Ui-Tei K (2009). siDirect 2.0: updated software for designing functional siRNA with reduced seed-dependent off-target effect.. BMC Bioinformatics.

[18] Reynolds A, Leake D, Boese Q, Scaringe S, Marshall WS (2004). Rational siRNA design for RNA interference.. Nat Biotechnol.

[19] Hunter RJ (1988).

[20] Kim D, Rossi J (2008). RNAi mechanisms and applications.. Biotechniques.

[21] Timmons L, Court DL, Fire A (2001). Ingestion of bacterially expressed dsRNAs can produce specific and potent genetic interference in Caenorhabditis elegans.. Gene.

[22] Gee L, Hartig J, Law L, Wittlieb J, Khalturin K (2010). beta-catenin plays a central role in setting up the head organizer in hydra.. Dev Biol.

[23] Hobmayer B, Rentzsch F, Kuhn K, Happel CM, von Laue CC (2000). WNT signalling molecules act in axis formation in the diploblastic metazoan Hydra.. Nature.

[24] David CN, Campbell RD (1972). Cell cycle kinetics and development of Hydra attenuata. I. Epithelial cells.. J Cell Sci.

[25] Holstein TW, Hobmayer E, David CN (1991). Pattern of epithelial cell cycling in hydra.. Dev Biol.

[26] Sproull F, David CN (1979). Stem cell growth and differentiation in Hydra attenuata. II. Regulation of nerve and nematocyte differentiation in multiclone aggregates.. J Cell Sci.

[27] Hobmayer B, Holstein T, David C (1990). Tentacle morphogenesis I.. Development.

[28] Hobmayer B, Holstein T, David C (1990). Tentacle morphogenesis in Hydra II.. Development.

[29] Yin X, Giap C, Lazo JS, Prochownik EV (2003). Low molecular weight inhibitors of Myc/Max interaction and function.. Oncogene.

[30] Huang MJ, Cheng YC, Liu CR, Lin S, Liu E (2006). A small-molecule c-Myc inhibitor, 10058-F4, induces cell-cycle arrest, apoptosis, and myeloid differentiation of human acute myeloid leukemia.. Exp Hematol.

[31] Gomez-Curet I, Perkins RS, Bennett R, Feidler KL, Dunn SP, Kruger LJ (2006). c-Myc inhibition negatively impacts lymphoma growth.. J Pediatr Surg.

[32] Galliot B, Miljkovic-Licina M, de Rosa R, Chera S (2006). Hydra, a niche for cell and developmental plasticity.. Semin Cell Dev Biol.

[33] Wittlieb J, Khalturin K, Lohmann JU, Anton-Erxleben F, Bosch TC (2006). Transgenic Hydra allow in vivo tracking of individual stem cells during morphogenesis.. Proc Natl Acad Sci U S A.

[34] Cardenas MM, Salgado LM (2003). STK, the src homologue, is responsible for the initial commitment to develop head structures in Hydra.. Dev Biol.

[35] Lohmann JU, Bosch TC (2000). The novel peptide HEADY specifies apical fate in a simple radially symmetric metazoan.. Genes Dev.

[36] Chera S, de Rosa R, Miljkovic-Licina M, Dobretz K, Ghila L (2006). Silencing of the hydra serine protease inhibitor Kazal1 gene mimics the human SPINK1 pancreatic phenotype.. J Cell Sci.

[37] Chera S, Ghila L, Dobretz K, Wenger Y, Bauer C (2009). Apoptotic cells provide an unexpected source of Wnt3 signaling to drive hydra head regeneration.. Dev Cell.

[38] Duffy DJ, Plickert G, Kuenzel T, Tilmann W, Frank U (2010). Wnt signaling promotes oral but suppresses aboral structures in Hydractinia metamorphosis and regeneration.. Development.

[39] Fire A, Xu S, Montgomery MK, Kostas SA, Driver SE (1998). Potent and specific genetic interference by double-stranded RNA in Caenorhabditis elegans.. Nature.

[40] Gheysen G, Vanholme B (2007). RNAi from plants to nematodes.. Trends Biotechnol.

[41] Timmons L, Tabara H, Mello CC, Fire AZ (2003). Inducible systemic RNA silencing in Caenorhabditis elegans.. Mol Biol Cell.

[42] Castanotto D, Rossi JJ (2009). The promises and pitfalls of RNA-interference-based therapeutics.. Nature.

[43] Kim DH, Rossi JJ (2007). Strategies for silencing human disease using RNA interference.. Nat Rev Genet.

[44] Whitehead KA, Langer R, Anderson DG (2009). Knocking down barriers: advances in siRNA delivery.. Nat Rev Drug Discov.

[45] Howard KA (2009). Delivery of RNA interference therapeutics using polycation-based nanoparticles.. Adv Drug Deliv Rev.

[46] Torchilin VP, Levchenko TS, Rammohan R, Volodina N, Papahadjopoulos-Sternberg B (2003). Cell transfection in vitro and in vivo with nontoxic TAT peptide-liposome-DNA complexes.. Proc Natl Acad Sci U S A.

[47] Saleh MC, van Rij RP, Hekele A, Gillis A, Foley E (2006). The endocytic pathway mediates cell entry of dsRNA to induce RNAi silencing.. Nat Cell Biol.

[48] de la Cova C, Johnston LA (2006). Myc in model organisms: a view from the flyroom.. Semin Cancer Biol.

[49] Wilson A, Laurenti E, Trumpp A (2009). Balancing dormant and self-renewing hematopoietic stem cells.. Curr Opin Genet Dev.

[50] Newmark PA, Reddien PW, Cebria F, Sanchez Alvarado A (2003). Ingestion of bacterially expressed double-stranded RNA inhibits gene expression in planarians.. Proc Natl Acad Sci U S A.

[51] Laurenti E, Wilson A, Trumpp A (2009). Myc's other life: stem cells and beyond.. Curr Opin Cell Biol.

[52] Laurenti E, Varnum-Finney B, Wilson A, Ferrero I, Blanco-Bose WE (2008). Hematopoietic stem cell function and survival depend on c-Myc and N-Myc activity.. Cell Stem Cell.

[53] Demeterco C, Itkin-Ansari P, Tyrberg B, Ford LP, Jarvis RA (2002). c-Myc controls proliferation versus differentiation in human pancreatic endocrine cells.. J Clin Endocrinol Metab.

[54] Gallant P (2009). Drosophila Myc.. Adv Cancer Res.

[55] Grens A, Gee L, Fisher DA, Bode HR (1996). CnNK-2, an NK-2 homeobox gene, has a role in patterning the basal end of the axis in hydra.. Dev Biol.

[56] Livak KJ, Schmittgen TD (2001). Analysis of relative gene expression data using real-time quantitative PCR and the 2(-Delta Delta C(T)) Method.. Methods.

[57] Sambrook J, Fritsch EF, Maniatis T (1989). Molecular Cloning: A Laboratory Manual, 2nd ed..

[58] David CN (1973). A quantitative method for maceration of Hydra tissue: Wilhelm Roux Arch.. EntwMech Org.

