# Supplementary figures and images for: Hymyc1 Downregulation Promotes Stem Cell Proliferation in Hydra vulgaris
Source: PLoS One. 2012 Jan 23;7(1):e30660. doi: 10.1371/journal.pone.0030660 (PMC3264606; doi:10.1371/journal.pone.0030660)

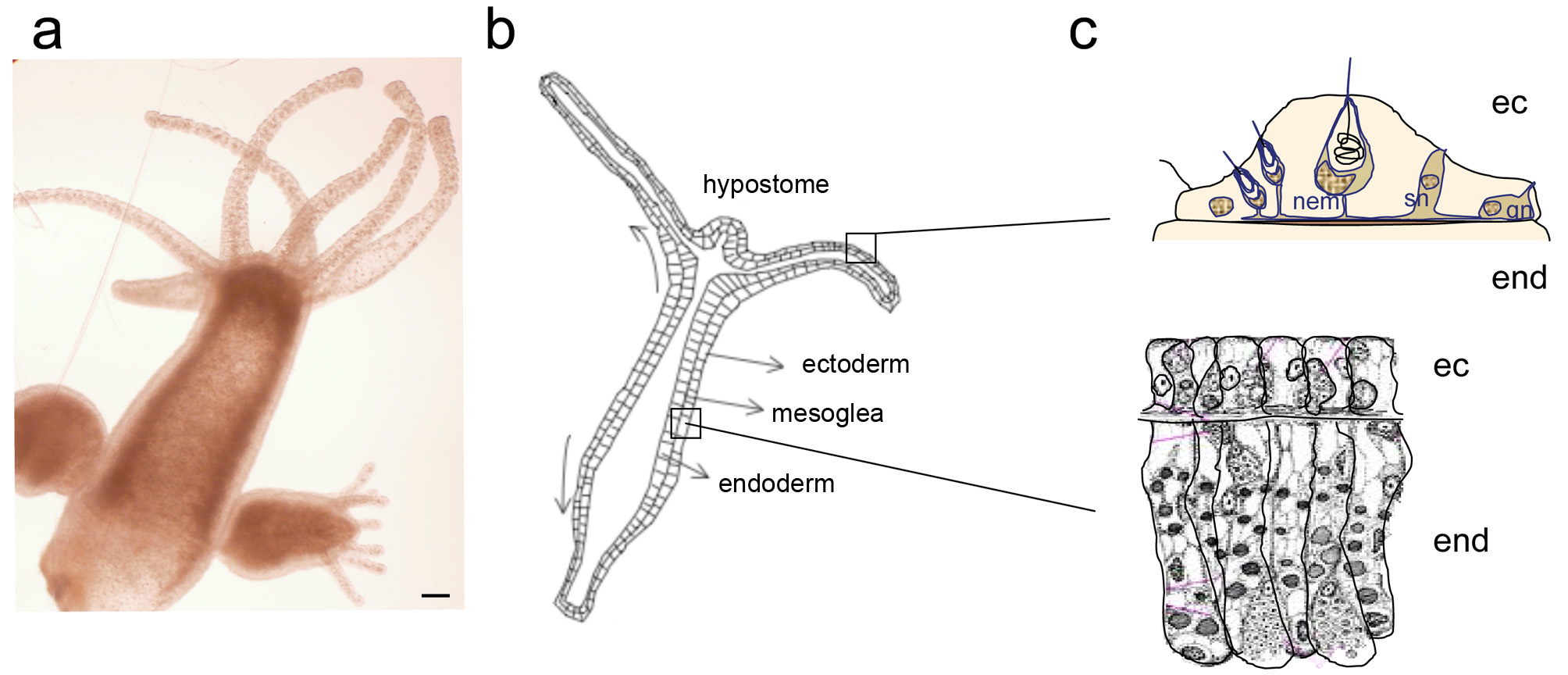

Supplement: Figure S1 — Structural anatomy of Hydra vulgaris . a) Picture of living Hydra. The animal has a simple body plan: it is a tube with a head at the apical end, and a foot, or basal disc at the other. The head is in two parts, the hypostome (mouth) at the apex, and below that the tentacle zone from which a ring of tentacles emerge. Scale bar 200 µm b) the bilayered structure of the animal: the body wall is composed of two self renewing cell layers, an outer ectoderm and an inner endoderm, separated by an extracellular matrix, the mesoglea. The arrows on the left side indicate the direction of tissue displacement c) longitudinal sections at level of tentacle (upper figure) and gastric region (lower figure). On the tentacle, the ectodermal cells (ec) are called battery cells and contain embedded several types of nematocytes (nem), one sensory neuron facing the exterior (sn), a ganglial neuron (gn) making connections both with other cells and to myonemes. Along the animal body both ectoderm and endoderm layers are composed of epitheliomuscular cells, while interstitial stem cells and their intermediate and terminal derivatives (neurons, nematocytes and secretory cells) are interspersed among ectoderm and endoderm. (TIF) [file pone.0030660.s001.tif]

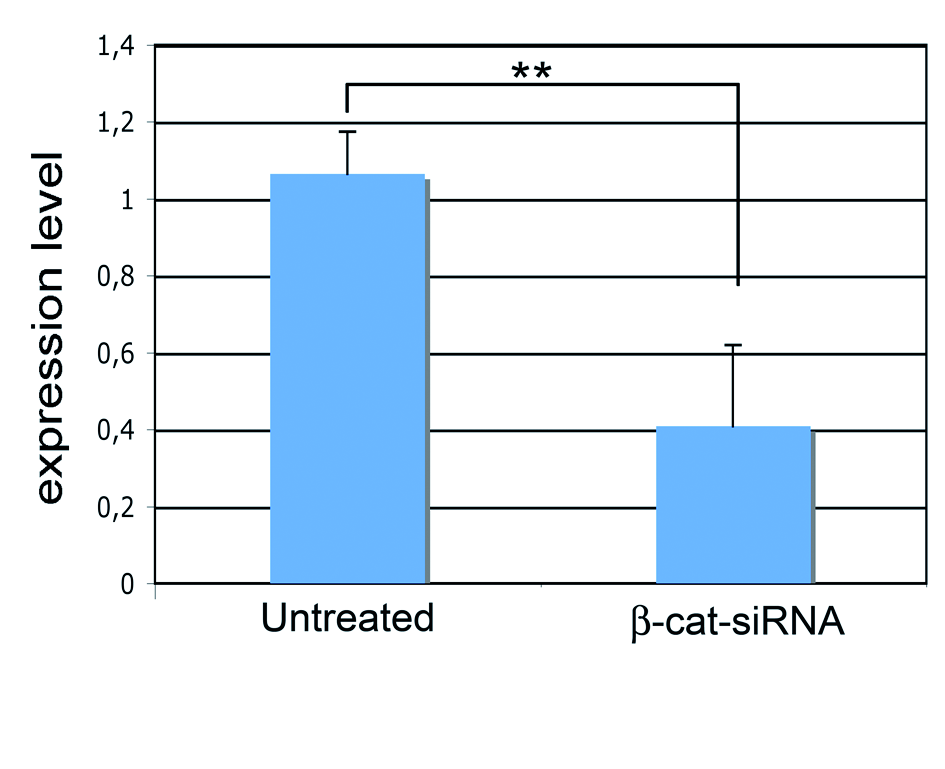

Supplement: Figure S4 — Efficient silencing of β-catenin gene through siRNA mediated RNAi. qRT-PCR was performed on total RNA extracted from polyps treated 2 d with Hydra β-catenin specific siRNA. A significant reduction of the Hyβ-cat transcript levels (60%), compared to HyEF1α, was induced by Hyβ-cat siRNA, showing the reliability and robustness of our approach. Two asterisks, p<0.01, according to t-Student test. (TIF) [file pone.0030660.s004.tif]

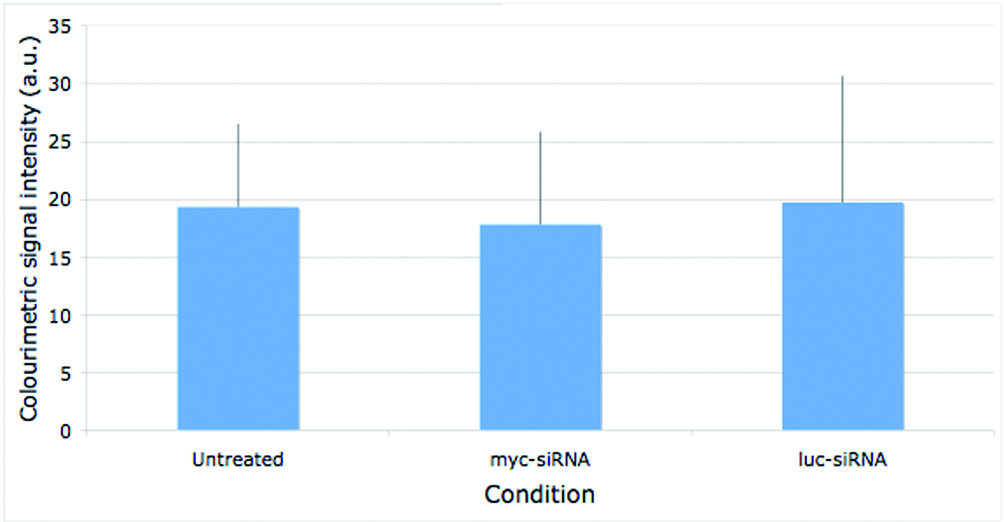

Supplement: Figure S5 — Quantification of in situ hybridization signal for Hymyc1 mRNA. The Image processing and Analysis software Image J (Version 1.45i) was used to quantify the signal intensity produced by in situ hybridization in control, myc-siRNA and luc-siRNA treated animals, using Hymyc1 as probe. Not significant differences were detected as effect of siRNA treatment, indicating residual Hymyc1 transcripts in myc-RNAi animals. On the other side, luc-siRNA animals were not affected in Hymyc1 expression, confirming the absence of putative side effects. (TIF) [file pone.0030660.s005.tif]

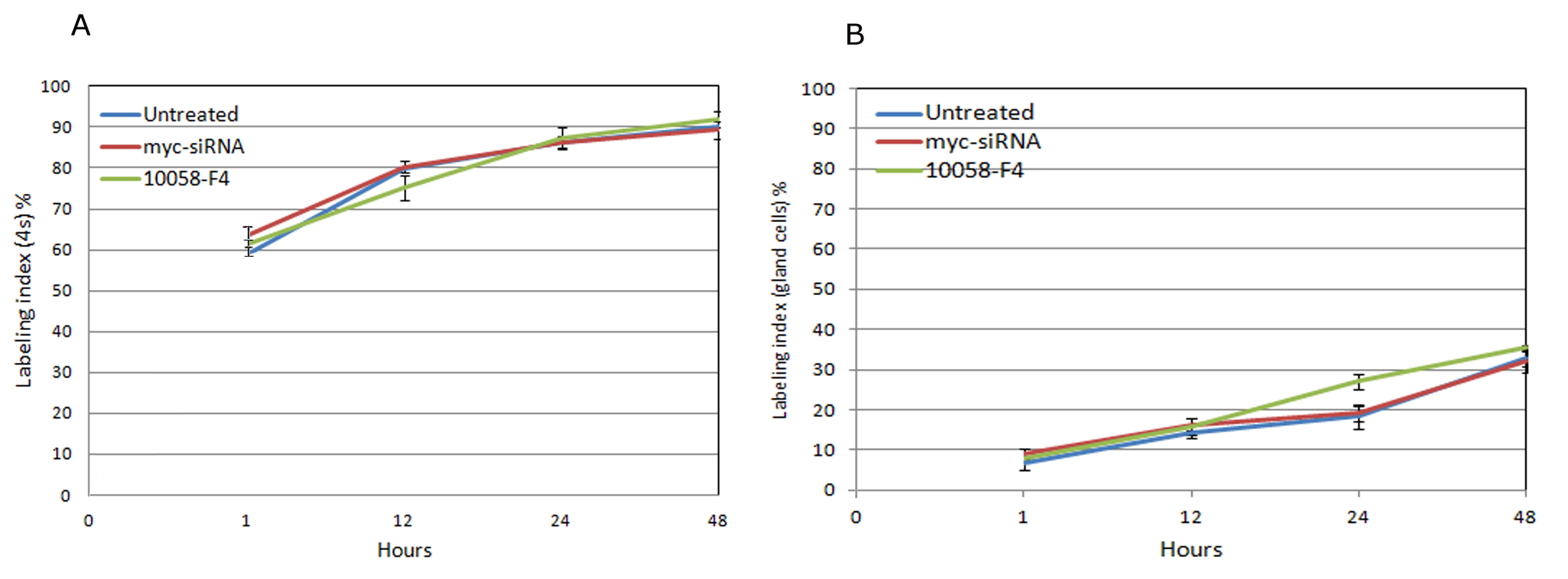

Supplement: Figure S6 — Effect of Hymyc1 RNAi on nematoblast and gland cell proliferation. Cell cycling activity of A) nematoblasts (4 s) and B) gland cells. Control untreated animals (incubated at pH 4) and myc-siRNA treated animals were continuously incubated with BrdU (red line) and with the c-myc inhibitor 10058-F4 (90 µM, green line), before maceration at the indicated time points and fluorescence immunostaining. Data represent the average of three different experiments. Not significant differences were observed in the proliferation rates of 4 s and gland cells induced by myc-siRNA or 10058-F4 treatments. (TIF) [file pone.0030660.s006.tif]

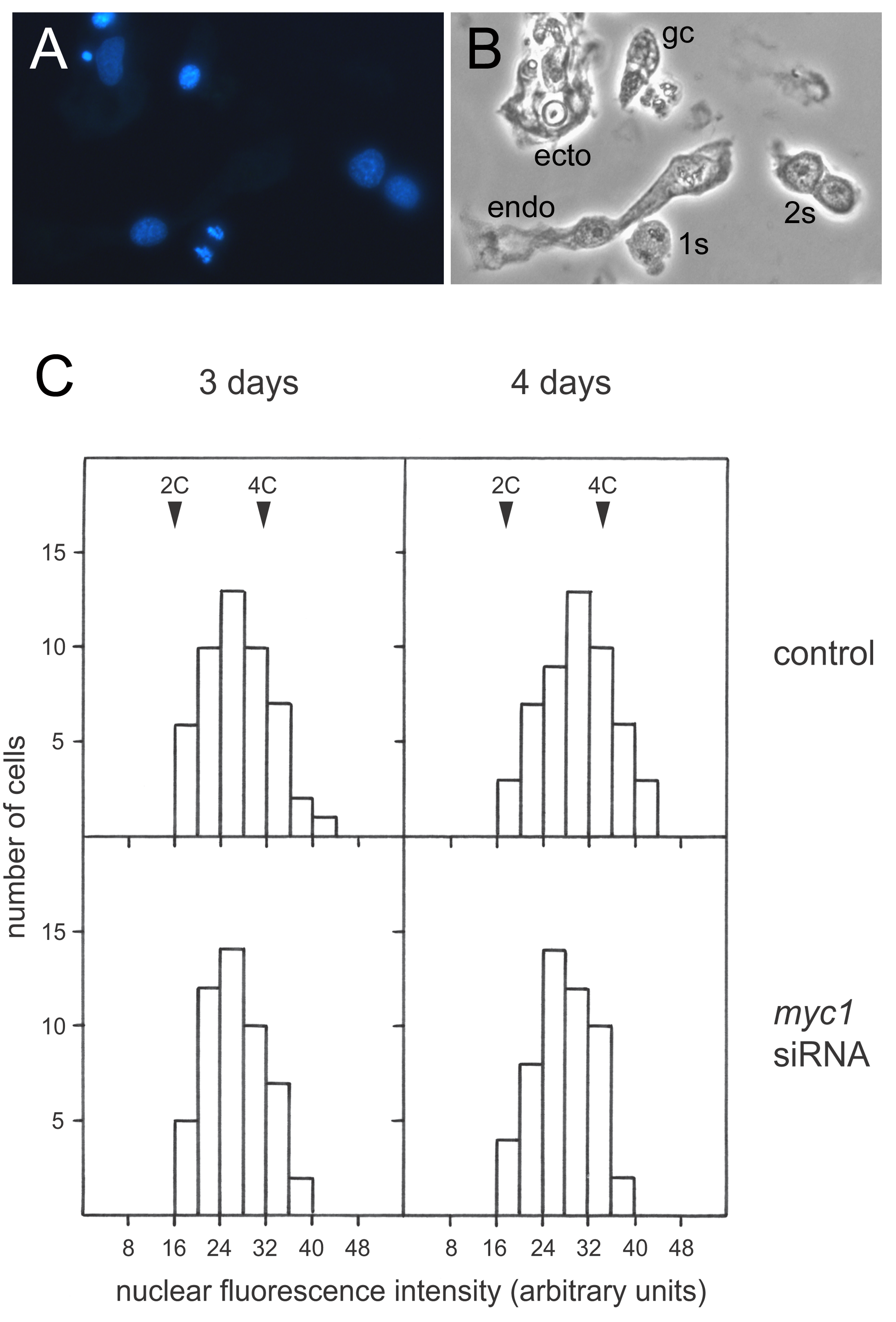

Supplement: Figure S7 — Nuclear DNA content of interstitial stem cells (1 s+2 s) in myc- siRNA treated Hydra . A) Hoechst 33342 staining and B) phase contrast image of macerated cells. 1 s and 2 s: large interstitial stem cells (note that 1 s is in mitosis); ecto: ectodermal epithelial cell; endo: endodermal epithelial cell; gc: gland cell. C) After three or four days of siRNA treatment, the nuclear DNA profile of 1 s+2 s shows no significant differences as compared with untreated polyps. Nuclei of 25 nerve cells and differentiated nematocytes, which are terminally arrested in G1, were used to determine the fluorescence intensity of the 2C DNA content. Procedures: Polyps were treated for three or four days with myc- siRNA. Then, treated and untreated animals were macerated and spread onto microscope slides. After drying, the maceration preparations were stained with Hoechst 33342. Fluorescence intensity of individual nuclei was finally quantified by using the Fiji program of the ImageJ software package. (TIF) [file pone.0030660.s007.tif]

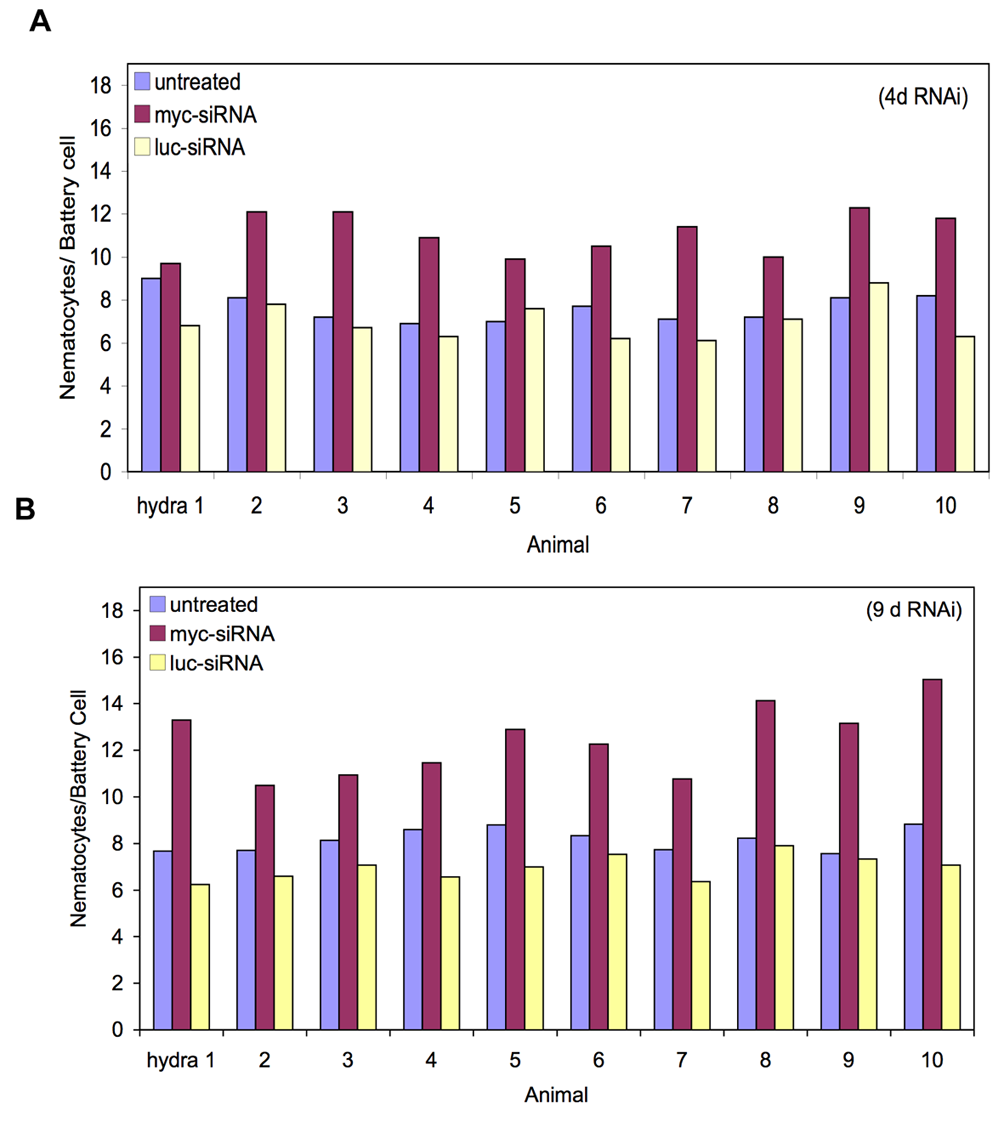

Supplement: Figure S8 — Distribution of the ratio nematocyte/battery cells among different animals. At time 4 d and 9 d of treatment with the indicated siRNA, animals were fixed and examined under an optical microscope. Under fixed focus plane, the ratio nematocyte/battery cell was calculated on a total of one hundred battery cells, collected on ten different polyps. myc-siRNA specifically induces an increase in the nematocytes embedded in the tentacle battery cells. (TIF) [file pone.0030660.s008.tif]
